# Elastofibroma Dorsi: Case Report with Point of Care Ultrasound Primary Care Applications 

**DOI:** 10.24908/pocus.v6i2.15184

**Published:** 2021-11-23

**Authors:** Trent Mazer, Karam Nabeel Gagi, Michael Bishop

**Affiliations:** 1 Department of Family Medicine, Mercy Health Grand Rapids, Michigan United States

**Keywords:** Elastofibroma Dorsi, Primary Care, Lipoma, Shoulder Pain

## Abstract

Elastofibroma dorsi (ED) is an uncommon, benign, slow-growing soft tissue tumor with an unclear etiology. The growth often presents as a local deformity with mild pain or discomfort in the subscapular region of geriatric populations. The following paper discusses a 73 year old female with mildly painful ED who presented to her primary care physician. We further review current literature on epidemiology, utilization of point of care ultrasound (POCUS) and treatment options.

## Introduction

Elastofibroma dorsi (ED) are rare, benign soft tissue tumors located most frequently between the inferior aspect of the scapula and the thoracic chest wall. ED were first described by Jarvi and Saxen in 1961 1. Since then, there have been multiple case reports and case series on these tumors which have broadened our understanding of their potential etiologies 2, 3, 4, 5, 6. The currently proposed mechanism for ED formation is a reactive process resulting from frictional irritation or trauma. Occasionally mucosal lesions have been reported, but are rare 7, 8, 9, 10, 11. 

The typical presentation of ED includes a history of a unilateral mass on the inferior aspect of the scapula that causes swelling and discomfort, and in rare cases, pain 12. On magnetic resonance imaging (MRI), the tumor is typically a solitary, poorly circumscribed, heterogeneous soft tissue mass 13.

## Case Presentation 

A 73-year-old female presented to her primary care provider due to worsening shoulder pain. On initial presentation the patient described intermittent pain localized in the right mid medial scapular region that was so significant she could not use her vacuum. This pain started about 6 weeks prior to presentation with the development of a mass the size of "half an orange" in the area of her bra line, near the right shoulder blade. She denied any trauma to that area and could not recall the mass development timeline. Medical history did include a right shoulder reverse arthroplasty. A quick review of her records showed that a previous computed tomography of the chest performed a year prior to surgery was referenced and found to have no abnormalities in the area of question. The initial visit was over the telephone due to the patient's COVID-19 concerns, thus no physical exam was completed at that time. 

**Figure 1:**
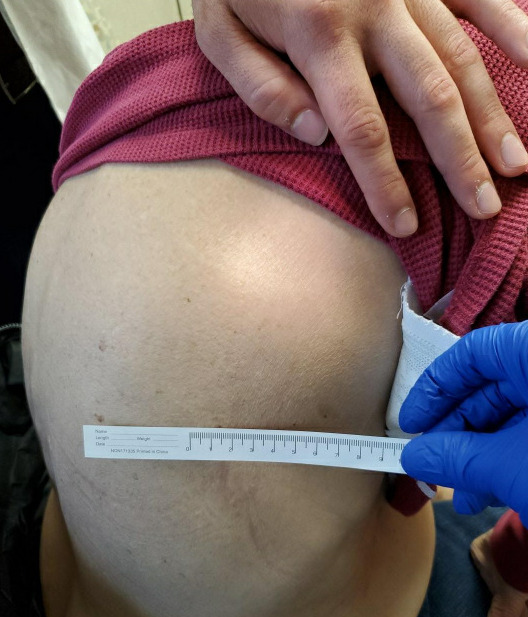
Picture of the Right posterior thorax. Visualization of the mass was possible from neutral positioning.

**Figure 2:**
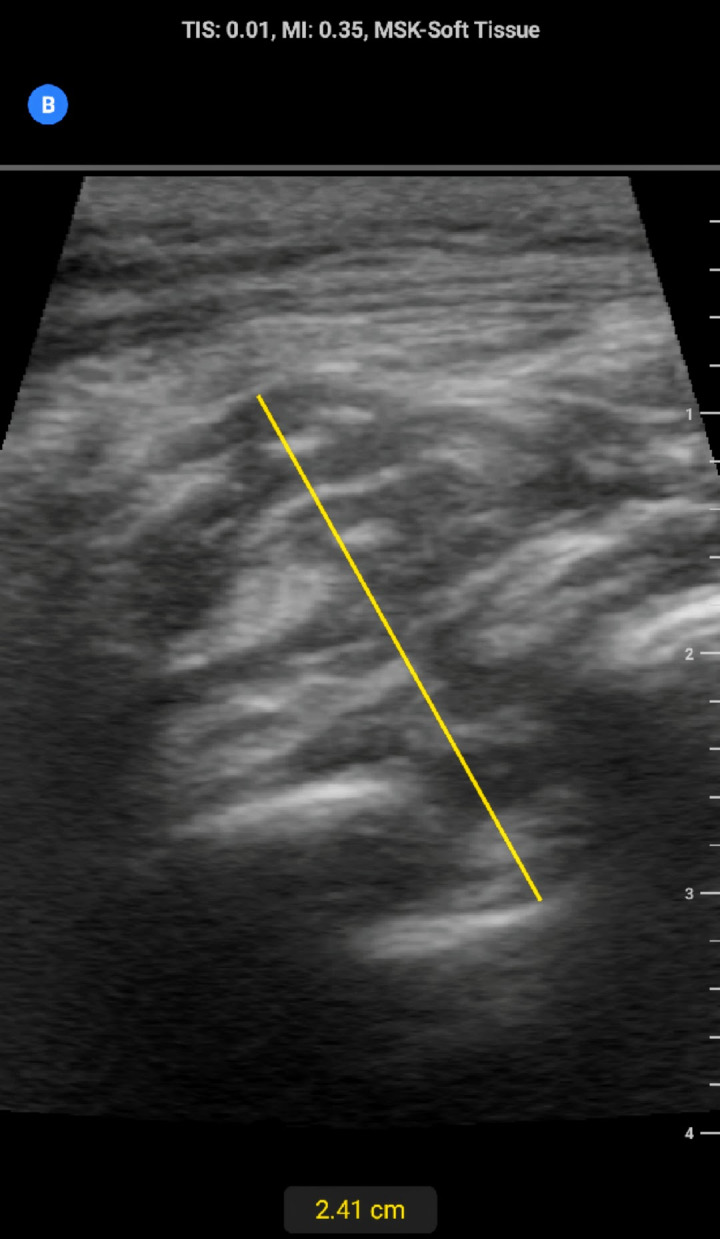
POCUS at initial office visit of the right posterior thorax below the scapula. Images include proximal long right lateral orientation just below the shoulder blade showcasing heterogeneous soft tissue mass located deep to the superficial musculature.

A month later at a subsequent encounter the mass was examined and found to be approximately 5 cm x 5 cm in diameter. It was visible in the neutral position, localized just inferior to the right scapula, nontender to palpation and nonmobile (Figure 1). Laboratory findings were within normal limits. POCUS revealed a mass that was deep to the latissimus dorsi and resting above the inferior portion of the scapula. The mass was heterogeneous, nonencapsulated with similar echogenicity to subcutaneous fat and interweaving hypoechoic regions indicating a possible inflammatory response with fluid buildup (Figure 2). Doppler was not utilized during this exam. 

**Figure 3:**
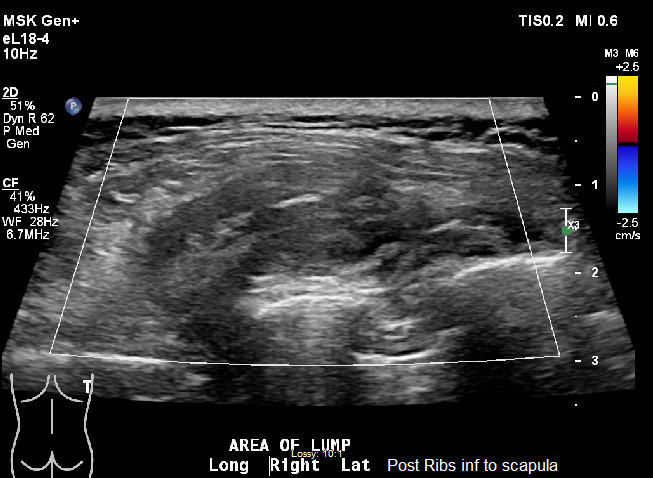
FormalUltrasound. Posterior right chest along the inferior aspect of the scapula superficial to the scapula and along the chest wall, a 4.8 x 4.1 x 1.9 cm heterogeneous soft tissue mass without significant flow seen on color Doppler imaging. It is located deep to the superficial musculature. Initial impression of a complex nonspecific soft tissue mass reflecting swelling from surgery or an injury. Neoplasm was not excluded.

**Figure 4:**
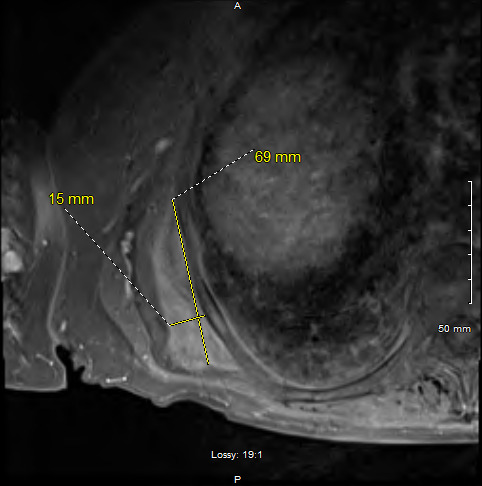
MRI of the Scapula. Mass deep to the right latissimus dorsi and serratus anterior muscles at and extending inferior to the distal tip of the scapula. This measures 6.9 x 1.5 cm, composed of soft tissue components of similar signal intensity in the skeletal muscle with small foci of intermixed fat and mild heterogeneous postcontrast enhancement. Impression of the exam was consistent with elastofibroma dorsi. [A:Anterior, S: Superior].

A formal ultrasound of the mass and chest Xray were ordered after the initial visit (Figure 3). The ultrasound completed a doppler examination that did not reveal any abnormal blood flow to the region. Results at this point were inconclusive and the primary care provider discussed the case with general surgery. An MRI was subsequently ordered and resulted in the diagnosis of ED (Figure 4.) The patient continued to have significant discomfort, thus surgical referral was placed for possible resection. 

## Epidemiology

ED is a rare, benign, slow growing soft tissue tumor that typically presents in the subscapular and infrascapular region 1. Some more rare locations of presentation include the orbit, mediastinum and greater omentum 14.

ED is most commonly found in the elderly, specifically over 55 years of age with a mean age of 60 years at diagnosis 12. Children have not been exempt with some literature finding cases in younger ages 14, 15, 16, 17, 18. Prevalence in the elderly ranges from 2% to 24% in women and 11% for men as reported in an autopsy series 15. 

The cause has not been determined, but ED is more common in people with large amounts of activity involving the shoulder 14, 15. This has led to the conclusion that increased friction between the scapula and the thoracic wall may be associated with the development of ED. Microtrauma is implicated to cause degeneration of collagen and reactive hyperproliferation of fibroblastic tissue in that region 15, 16. It is difficult to explain the development of ED in sites not involved in mechanical overload leading authors to consider ED as more of a normal aging process or genetic predisposition 14, 17.

## Imaging Applications

**Figure 5:**
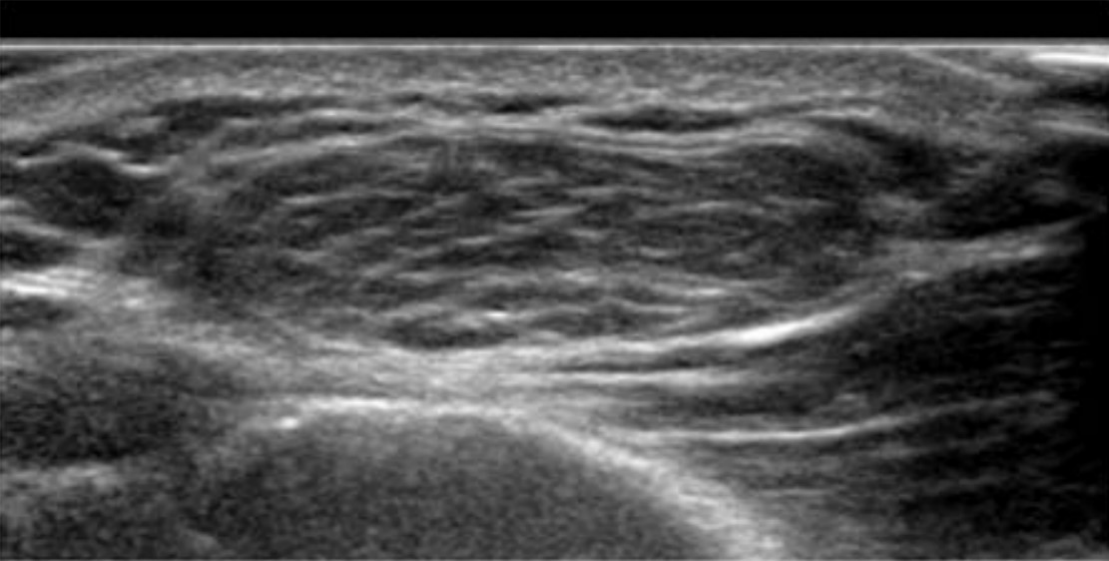
Example of benign lipoma with sonographic appearance of well organized, heterogeneous echogenicity with multiple clearly defined long smooth continuous internal echogenic lines parallel to the long axis of the lesion superficial to any musculature 20.

Ultrasound examination as a screening test can quickly identify masses with concerning features such as a diameter larger than 5 cm, location below the muscle, heterogeneity and increased doppler flow 13. Figure 5 can be referenced as an example of a benign soft tissue mass for comparison. The decision to move towards definitive diagnosis with an MRI could be expedited in concerning cases if POCUS was used in the primary care setting. Avoiding the distress and service demands of unnecessary urgent cancer referrals 13, 14. In addition primary care POCUS has been found to be a reliable tool in lipoma evaluations with proper training 18. On re-evaluation of the initial POCUS images in this case, the heterogenous, disorganized structure underneath the superficial muscle that was increasing in size and pain could have led directly to MRI evaluation. The official ultrasound and radiography offered no further diagnostic clues beyond a negative doppler, and in this case could have been bypassed for surgical evaluation or MRI since the diagnosis was uncertain 19, 12, 13. 

## Treatment

Upon definitive diagnosis of ED, treatment depends on severity of symptoms. Asymptomatic patients with ED do not benefit from excision as ED is a benign process. Clinical follow up proves to be adequate for this population 5, 21. When ED causes significant symptoms for the patient or the diagnosis is not definitive, curative marginal resection is recommended 5, 19. No cases of malignant transformation have been recorded and beyond incomplete excision, recurrence is rare 16, 19, 22, 23. 

In conclusion, understanding the presentation of ED could help patients avoid unnecessary procedures. The elderly and asymptomatic patients are most at risk, and simple follow up is most often sufficient. Only those who are symptomatic should be provided the opportunity to proceed with surgical management. 

## Declaration of patient consent

The authors certify that they have obtained all appropriate patient consent forms. In the form the patient has given her consent for her images and other clinical information to be reported in the journal. The patient understands that her name and initials will not be published and due efforts will be made to conceal her identity, but anonymity cannot be guaranteed.

## Disclosures

None.
